# Supplementary figures and images for: Impact of suboptimal donor to suboptimal recipient kidney transplant on delayed graft function and outcome
Source: Front Transplant. 2023 Sep 12;2:1240155. doi: 10.3389/frtra.2023.1240155 (PMC11235345; doi:10.3389/frtra.2023.1240155)

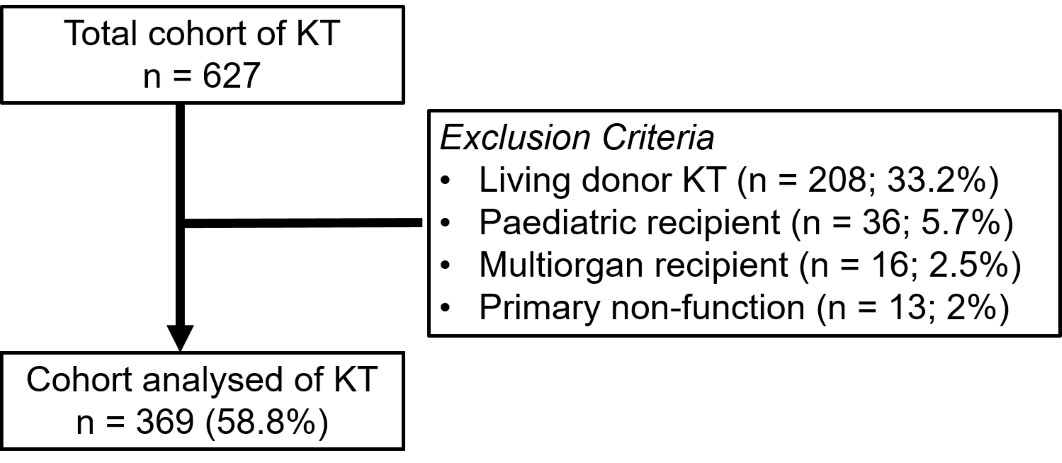

Supplement: Supplementary file 2 [file Image1.jpeg]

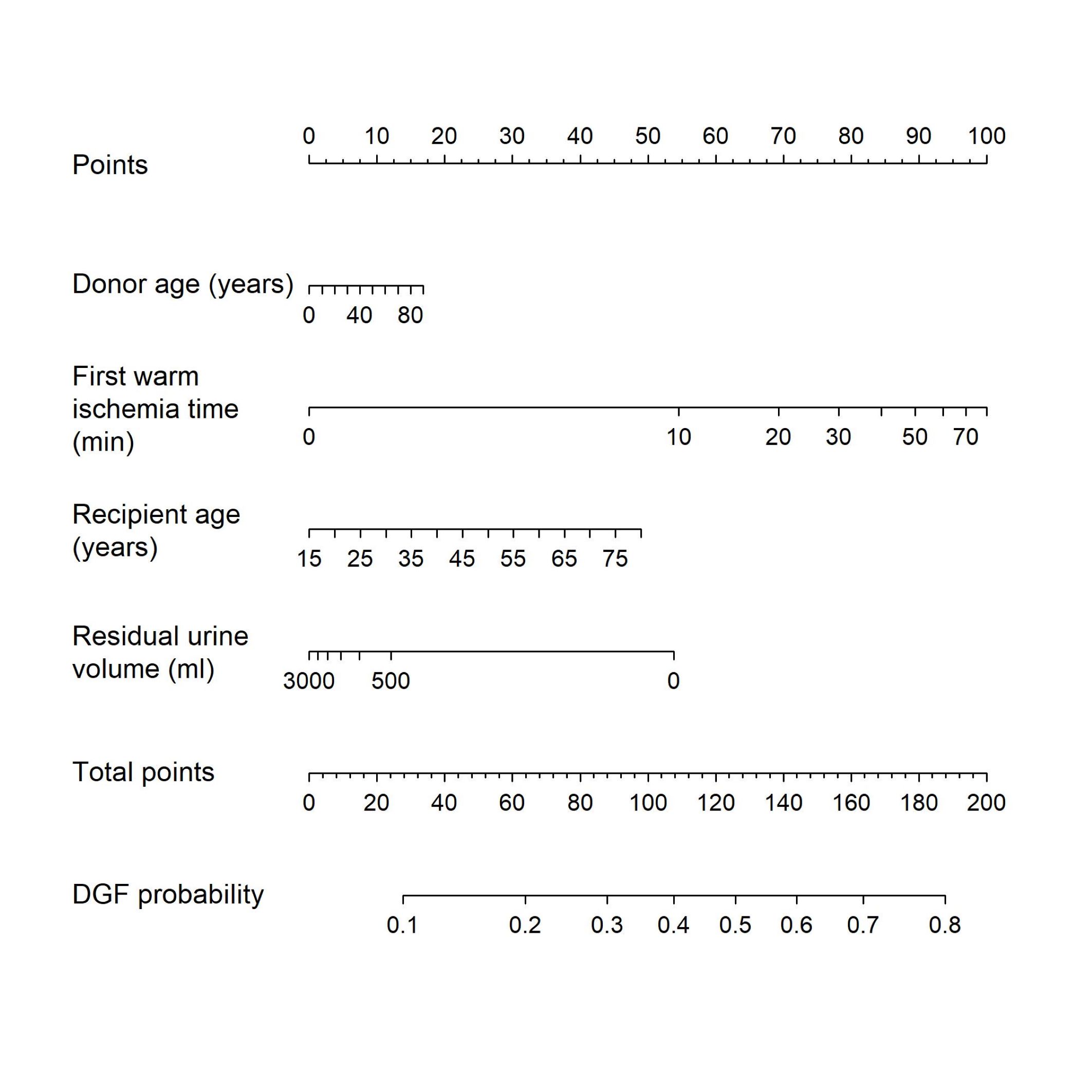

Supplement: Supplementary file 3 [file Image2.jpeg]
